# Induced Pluripotent Stem Cells: Advances in the Quest for Genetic Stability during Reprogramming Process

**DOI:** 10.3390/ijms18091952

**Published:** 2017-09-13

**Authors:** Valentina Turinetto, Luca Orlando, Claudia Giachino

**Affiliations:** 1 Department of Clinical and Biological Sciences, University of Turin, Regione Gonzole 10, 10043 Orbassano, Turin, Italy; valentina.turinetto@unito.it; 2 McMaster Stem Cell and Cancer Research Institute, Michael G. DeGroote School of Medicine, McMaster University, Hamilton, ON L8S 4L8, Canada; lorland@mcmaster.ca

**Keywords:** DNA damage response, genetic stability, genetic variation, induced pluripotent stem cell, reprogramming

## Abstract

Evaluation of the extent and nature of induced pluripotent stem cell (iPSC) genetic instability is important for both basic research and future clinical use. As previously demonstrated regarding embryonic stem cells, such DNA aberrations might affect the differentiation capacity of the cells and increase their tumorigenicity. Here, we first focus on the contribution of multiple DNA damage response pathways during cellular reprogramming. We then discuss the origin and mechanisms responsible for the modification of genetic material in iPSCs (pre-existing variations in somatic cells, mutations induced by reprogramming factors, and mutations induced by culture expansion) and deepen the possible functional consequences of genetic variations in these cells. Lastly, we present some recent improvements of iPSC generation methods aimed at obtaining cells with fewer genetic variations.

## 1. Introduction

Induced pluripotent stem cells (iPSCs) hold equivalent embryonic stem cells (ESC) properties (self renewal and ability to differentiate into all three embryonic germ layers) [1,2,3] and are great promise for clinical applications because of their potential use in personalized cell therapy or disease modeling [4]. To achieve these functions, it is fundamental that they possess an intact and stable genome. Much research has been performed in the field of genome maintenance in mouse and human ESCs. However, less is known about the causes of genomic aberrations and the functionality of repair mechanisms in iPSCs [5]. In this review, we will address the contribution of multiple DNA damage response (DDR) pathways during cellular reprogramming, considering both mouse iPSCs (miPSCs) and human iPSCs (hiPSCs). Then, we will discuss about the origin and mechanisms responsible for the modification of genetic material, focusing on hiPSCs. We will also deepen the possible functional consequences of genetic variations in hiPSCs. Lastly, we will present some recent improvements of hiPSC generation methods aimed at obtaining cells with fewer genetic variations.

## 2. Cell Reprogramming Is a Process That Involves DNA Damage and Efficient Repair Mechanisms

iPSCs share numerous similarities in DDR with ESCs, including G_2_/M cell cycle arrest, efficient double strand break (DSB) repair, and high expression of DNA damage signaling and repair genes [6]. However, DDR pathways are also widely involved during the reprogramming process, due to its dramatic effect on chromatin structure rearrangement and genetic expression.

The acceleration of growth rate following the induction of reprogramming factors imposes greater metabolic demands for energy. This is accomplished through metabolism shifts from oxidative respiration to oxidative glycolysis in both mouse and human iPSCs [7,8]. Such a metabolic shift can lead to a buildup of electrons in the electron transport chain, and this increases their leakage into the cytoplasm as reactive oxygen species (ROS) that will cause oxidative stress. High ROS levels can modify individual nucleotide bases, as is the case for the mutagenic 7,8 dihydro-8-oxoguanine, can cause the production of single- and double-strand breaks [9], and can result in telomere attrition [10]. As result, reprogramming-factor-transduced fibroblasts have elevated levels of oxidative DNA damage and ROS [11,12]. A second consequence of growth rate acceleration is replication stress promotion that is characterized by elevated numbers of stalled and collapsed replication forks, leading to DNA damage. Unreplicated or unresolved regions create a physical link between sister chromatids and restrain chromosome segregation, determining the formation of anaphase bridges and accounting for replication-induced chromosomal instability [13]. At variance with somatic cells that activate checkpoints to prolong mitotic arrest in presence of incomplete DNA replication, pluripotent stem cells fail to generate the single-stranded DNA regions in response to replication stress, necessary for checkpoint activation and initiation of homologous recombination (HR) repair initiation. [14]. Another suggestion of iPSCs exceptional vulnerability to replication stress is the observation that recurrent aneuploidies lead to augmented proliferation and sensitivity to replication inhibitors [5,15,16].

Given that cellular reprogramming is associated with the generation of DNA DSBs, the most serious form of DNA damage, it is not surprising that the generation of iPSCs requires the engagement of multiple DDR pathways, independently of vector integration or use of non-integrating reprogramming strategies. Both non-homologous-end-joining (NHEJ) and HR, the two main cellular DSB repair pathways [17], have been found to be involved in reprogramming. Gonzalez et al. [18] showed that ectopic expression of the reprogramming factors was sufficient to induce DNA DSBs in mouse iPSCs cells. Furthermore, they showed that efficient reprogramming required key HR genes, including *Brca1*, *Brca2* and *Rad51* [18]. These data suggested that an intact HR pathway is required to achieve efficient reprogramming, even in the absence of potential genome modifying agents such as the oncogene *c-Myc* or viral-integration [18]. In accordance with these data, another paper demonstrated the key role of *Rad51* in the reprogramming process [19]. Co-expression of *Rad51* with *Oct4*, *Sox2*, *Klf4*, and *c-Myc* significantly increased iPSC generation efficiency, by regulating HR pathway during the early phases of the reprogramming process [19]. Tilgner et al. further reported a significant decrease in reprogramming efficiency and accumulation of chromosomal abnormalities in cells deficient for the DNA Ligase IV (*LIG4*) gene, which is essential for performing the final “end-joining” step of NHEJ [20]. The same group reported impaired reprogramming efficiency in cells bearing mutations in the *XLF* (*LIG4* cofactor, Cernunnos/*XLF*) gene, a key factor involved in the end-joining step of DNA repair during NHEJ process [21]. The important contribution of both *LIG4* and *DNA-PK* genes in reprogramming process was also reported by another group [22].

Other evidence of the importance of DDR pathways is the observation that deficiency of Ataxia-telangiectasia mutated (ATM), a protein kinase that has a critical role in the response to DNA double strand breaks [23], decreases reprogramming efficiency and increases genomic instability in mouse iPSCs [24]. Moreover, in the absence of a functional Fanconi Anemia (FA) pathway, caused by mutations in genes regulating replication-dependent removal of interstrand DNA crosslinks and responsible for the inherited genomic instability disorder FA [25], the attempts to obtain iPSC-like colonies were unsuccessful [26].

For an efficient reprogramming, a functioning nucleotide excision repair (NER) is also required. A recent work investigated the possibility to generate iPSCs from patients with Xeroderma pigmentosum (XP), a disease that exhibits NER deficiency [27]. Authors observed that iPSCs from cells defective in the *XPA* gene were generated with a lower efficiency in comparison to control cells. Additionally, XP-iPSCs exhibited hypersensitivity to ultraviolet exposure and accumulation of single-nucleotide substitutions [27].

The reason for different DDR pathway involvement in cell reprogramming is likely to avoid presence of aberration from the process itself or from the cells of origin. Marion and colleagues [28] showed in fact that reprogramming is limited in mouse and human iPSCs to prevent genomic istability by a p53-mediated DNA damage response that involves the activation of DSB response machinery, including histone variant H2A.X phosphorylation (γH2A.X). γH2A.X, one of the most characterized events involved in DSB response and a robust marker for DNA-DSBs, plays a critical role in iPSC generation. Increased γH2A.X level was reported during mouse embryonic fibroblast reprogramming, without any correlation with viral integration [18]. Moreover, γH2A.X and 53BP1 foci were reported to increase during fibroblast reprogramming and during long-term iPSC in vitro culturing, in comparison to the fibroblasts from which they derived [29]. Interestingly, the rate of H2A.X histone deposition pattern has been recently demonstrated to represent a functional marker for iPSC quality assessment [30], further supporting the important roles for H2A.X and its phosphorylation in the pluripotent state in addition to the canonical role in DSB response [31,32]. Since DDR pathways have been shown to be widely involved in the reprogramming process, it is not surprising to note that their defects are linked to genetic instability in iPSCs, owing to inefficient DNA repair and/or the preferential use of error-prone mechanisms.

These observations highlight that iPS reprogramming involves DDR machinery activation and that an efficient repair mechanism is needed to allow successful cell reprogramming.

## 3. Genetic Variations Identified in Human iPSCs

Notwithstanding the efficient DDR activation which occurs during reprogramming, de novo genetic variants in iPSCs have been observed in many studies [33,34,35,36,37,38,39] using both conventional methods and high-throughput technologies such as next-generation sequencing (Table 1). Overall, results illustrated the dynamic nature of genomic abnormalities in iPSCs and the consequent need for frequent genomic monitoring to assure phenotypic stability and clinical safety [37]. A wide range of variations have been identified so far in iPSCs, including chromosomal aberrations and aneuploidy, sub-chromosomal copy number variations (CNVs), and single nucleotide variations (SNVs).

### 3.1. Chromosomal Instability

The first attempt of a systematic identification and classification of hiPSCs chromosomal aberrations has been reported by Mayshar et al. [34]. They performed global gene expression meta-analysis on sixty-six hiPSC samples from several different studies to detect full and partial chromosomal aberrations [34]. This experimental approach is justified by the increasing evidence that genes that reside together on a chromosome show increased or decreased expression when a genomic alteration has occurred. Considering the origin of these aberrations, some were probably originated from the parent somatic cells, others derived from in vitro culturing; finally, another group of aberrations, evident in early passage hiPSCs, were presumed to occur during the reprogramming process [34]. Chromosome 12 trisomy is the most frequent chromosomal aberration identified in hiPSCs; notably, both Nanog homeobox (*NANOG*) and Growth Differentiation Factor 3 (*GDF3*), which are implicated in pluripotency maintenance, reside on chromosome 12p and are overexpressed consequently. Furthermore, several other genes that are involved in cell cycle regulation were reported to be expressed as a consequence of iPSC genomic aberrations [34]. This finding was later confirmed by two other individual studies [37,40] and one large-scale study [41]. It is interesting to note that gain of 12p is also found in testicular germ cell cancer [46,47]. Taapken et al. performed karyotype analysis on >1700 human iPSC and ESC cultures collected in almost 30 different laboratories without revealing notable differences in the incidence of chromosomal aberrations in iPSCs and ESCs [41]. Trisomy 12 was the predominant abnormality in both cell types, representing 42.6% of total aberrations detected in ESCs and 31.9% in iPSCs. Trisomy 8 occurred more frequently in iPSCs than in ESCs, partial gain of chromosome 12 and trisomy 20q were recurrent in both iPSC and ESC lines and an added X chromosome, recurrent in female ESC lines, occurred only in one iPSC line. Trisomy 17 was described in ESCs, but was never detected in iPSCs [41]. The frequency and types of karyotypic abnormalities are not affected by the method used to reprogram iPSCs or the substrates on which ESCs or iPSCs were cultured. While recurrent translocations are associated with specific cancers, they found no recurrent translocations in their large data set [41].

These data suggest that, in general, human iPSCs and ESCs share similar chromosomal instability, and that it is mainly a result of cell culture selective pressure and is independent of reprogramming procedure.

### 3.2. Copy Number Variations

Using array comparative genomic hybridization (array CGH), Chin et al. conducted the first CNV analysis of hiPSCs [48]. They analyzed three hiPSC lines and the fibroblasts from which they were derived and found a few CNVs in each iPSC line tested, yet none were shared between iPSC lines [48], suggesting the possibility that these CNVs may be acquired during reprogramming or in early culture of the hiPSCs lines. Later, a wider study was performed, monitoring the genomic stability of 32 hiPSC lines in array CGH [40]. These data highlighted that CNVs in iPSCs were two-fold higher than the levels present in their parental fibroblast cells or in ESCs. Authors identified unique CNV signatures for hiPSCs derived from specific sources of parental fibroblasts. A first group of CNVs included CNVs that may have a somatic origin, being shared between hiPSCs and their respective parental fibroblasts. A second group included CNVs detected only in hiPSCs and not in the parental fibroblasts, suggesting that may have been acquired during reprogramming or early culture of the hiPSC lines. They also identified recurrent CNVs at 1q31.3, 8q24.3 and 17q21.1 that are unique to the hiPSC samples (>25% of hiPSC samples) and are recurrently acquired during prolonged periods in culture. Other CNVs that were detected at high incidence in the hiPSC samples included duplications of 20q11.21 (18%) and 2p11.2 (>25%), which are also recurrently acquired in hESCs [42]. Overall, they identified CNVs that are unique to hiPSCs regardless of the tissue source and derivation methods, and CNVs shared by both hiPSCs and hESCs. Interestingly, an amplification of 20q11.21 was identified as among the most recurrent CNVs hotspot in hiPSCs (as well as hESCs) in several papers [37,40,43] and it was also frequently found in cancer samples [49,50]. Notably, this region is enriched with genes associated with pluripotency and apoptosis resistance. In another interesting work, Laurent et al. compared the relative number, length and distribution of CNVs among hESCs, hiPSC, and non-PSCs [37]. Overall, iPSCs were characterized by a significantly higher number of regions of duplication and deletion, with a distribution different to that observed in hESCs [37]. They found that CNVs were more abundant within pluripotency-associated genes and were affected by culture length and conditions. Prolonged in vitro culturing of hiPSCs favored duplications of oncogenic genes, whereas reprogramming was linked to tumor-suppressor gene deletions. Interestingly, they found a high frequency of duplications in pseudogenes of the pluripotency-associated *NANOG* and *OCT4/POU5F1* genes, including *NANOGP1*, that, similar to other genes active in early embryogenesis, tend to have many pseudogenes [51]. Up to now, it is not known if transcribed pseudogenes can influence in some ways the cellular function, however it is possible to hypothesize that the pseudogenes of pluripotency-associated genes regulate these genes in a positive or negative way. A separate study highlighted that CNVs in iPSCs were two-fold higher than the levels present in their parental fibroblast cells or in ESCs [36]. Hussein and colleagues showed that CNVs form at a high rate during reprogramming (possibly owing to replication stress), however, during propagation, rapid selection occurs against cells with high numbers of CNVs. In this way, it seems that much of the de novo CNVs render iPSCs selectively disadvantaged. However, it is possible that some CNVs could confer a selective advantage.

Collectively, these data suggest that CNVs occur more frequently in hiPSCs in comparison to ESCs and most of recurrent CNVs described in hiPSC are not present in hESC, suggesting that CNVs may have a somatic origin and that the reprogramming program could favor their occurrence.

### 3.3. Single Nucleotide Variants

Using high-throughput next generation sequencing analyses, an average of ten protein-coding mutations per hiPSC line were identified [33,35,44,45,52]. Gore and colleagues looked for point mutations performing exome sequencing on 22 hiPSC lines, generated in seven laboratories by five different methods, and their parental fibroblasts. They validated 124 mutations with capillary sequencing, which revealed that each mutation was fixed in heterozygous condition in the hiPSC lines [33]. From these data, they predicted a mutational load of six coding mutations per iPSC genome, independent of the reprogramming technology used (integrative versus non-integrative methods). Similarities between the reprogramming-associated mutational load and the process of malignant transformation were pointed out, as many of the identified missense mutations were predicted to alter functionality of proteins; however, the identified mutations were not analyzed experimentally in this work to assess their true functional significance. Through ultradeep sequencing, authors found that around half of the mutations pre-existed at low levels in the fibroblast populations and the others occurred during or after reprogramming [33]. Furthermore, the observation of mutated genes involved in human Mendelian disorders suggested that the risk for diseases other than cancer would need to be evaluated for hiPSC-based therapeutic methods [33]. On the other hand, Ji et al. performed whole exome sequencing of several iPSC lines at two different passages to determine the iPSC mutation rate and to assess the presence of mutations in *TP53* [44], considering its central role in maintaining iPSC genome integrity [28]. Their data concerning mutation rate highlighted that reprogramming contributes approximately 75% of the mutations found in the fibroblast-derived iPSCs and only a low percentage of mutations was preexisting in the parental cells. This reduced contribution of parental fibroblasts to iPSC mutations is in contrast with what reported in Gore’s and colleagues’ paper. However, it is important to note that Ji et al. characterized iPSCs derived from neonatal fibroblasts, a source expected to have less mutation accumulation compared to adult fibroblast used in Gore’s study. Further analysis of TP53 locus showed that none of its 11 exons had non-synonymous mutations and there were also no deleterious variants in *MDM2*, *CDKN2A*, *P21*, and *BCL2*, which are genes upstream or downstream of p53 in any of the iPSC lines analyzed. [28]. Thus, the iPSC line does not have obvious defects in genome maintenance that may make it prone to incur mutations during reprogramming. As the above studies had focused on fibroblast-derived hiPSCs, in order to assess if alternative cell sources would guarantee increased genetic stability based on their different reprogramming efficiencies, Ruiz et al. characterized the genomic integrity of eight hiPSC lines derived from five different non-fibroblast somatic cell types [45]. They showed that protein-coding mutations were a general feature of the hiPSC state and were independent of somatic cell source [45].

These presented studies highlighted that the different iPSC lines examined did not share any of the described SNVs, suggesting the stochastic nature of iPSC generation and mutations during reprogramming. One single study showed that all selected iPSC clones, in one of the reprogramming experiments, shared genetic variants which could also be detected in a rare subpopulation of the parental fibroblast pool; this suggested that cloning of individual source cells might be favored by their genetic composition leading to preferential reprogramming into iPSCs [53].

To date, there are no similar investigations on hESCs, rendering it more difficult to elucidate how SNVs occur. From the published results, it seems that a certain quote of SNPs derives from the original somatic cells and another occurs during or after reprogramming. It will be relevant to consider possible functional effects derived from mutations that are predicted to alter protein functions.

## 4. Sources of Genetic Variations in iPSCs

**Figure 1:**
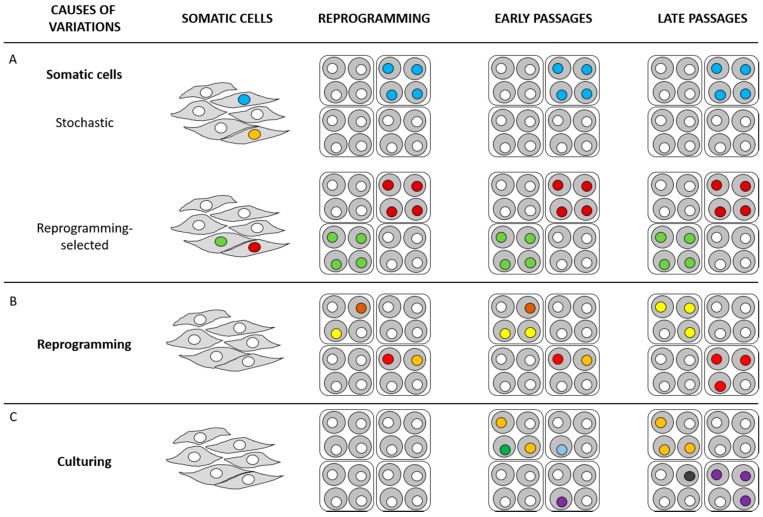
Origin of genetic variations in human induced pluripotent stem cells (hiPSCs). Genetic variations of hiPSCs may have at least three origins. (**A**) Pre-existing variations in parental somatic cells. If a pre-existing genetic variation does not influence the reprogramming process, iPSC generation can occur in a stochastic manner; in this way, it is possible that the cell with the genetic variation highlighted as a blue nucleus progresses in reprogramming, while the cell with the genetic variation highlighted as an orange nucleus does not generate any iPSC clone (stochastic). Differently, if a pre-existing genetic variation confers an advantage in the reprogramming process (garnet and green nuclei), reprogramming preferentially takes place in these cells (reprogramming-selected). (**B**) Reprogramming-induced mutations that occur during the reprogramming process. Mutations conferring advantages in self-renewal and/or proliferation eventually prevail the culture (red and yellow nuclei); mutations being deleterious for cell survival are selected against in culture (brown and orange nuclei). (**C**) Passage-induced mutations that arise during the prolonged culture. Analogously to the mutation selection described in (**B**), some mutations can prevail in culture (purple and orange nuclei), while others can be selected against in culture (green and sapphire blue nuclei). Prolonged culture can promote the appearance of further mutations in late passage (black nucleus). White nuclei represent cells with no genetic variations.

Data presented in the previous section suggest that, while chromosomal instability appears to derive mainly from cell culture conditions, CNVs and SNPs seem to be mainly related to the original somatic cell genetic background and/or the reprogramming processes. Researchers came to these conclusions through the comparison between different iPSC lines, their original somatic cells and ESC lines. Some more recent papers tried to face the issue regarding the sources of genetic variations in iPSCs through specific and/or innovative approaches (Figure 1) [39].

### 4.1. Pre-Existing Genetic Variations in Parental Cells

As previously discussed, studies highlighting genetic variations in iPSCs originating from parental somatic cells are not concordant on the reported frequency [33,40,44]. Reasons may include technical difficulties in the identification of pre-existing mutations that can be overtaken by the application of ultra-deep sequencing for the identification of low frequency variations that may pre-exist in parental somatic cells. To overcome these difficulties in determining whether iPSCs are inherently more likely to accumulate mutations and further elucidate the origin of genomic variants present in iPSCs, a very recent study applied a new approach by deriving fibroblast sub-clones and clonal iPSC lines from the same fibroblast population and applied next-generation sequencing to directly assess whether the reprogramming process leads to more mutations [54]. These data showed that iPSCs did not contain more genomic variations than the fibroblast sub-clones, suggesting that the iPSC reprogramming is not mutagenic. In addition, more than 90% of the putative new mutations in both daughter lines preexisted in the parental fibroblast population at very low frequencies as mosaic variants. Only a small number of variants remained undetectable in the parental fibroblasts, which were thus likely to be de novo. Importantly, the clonal iPSCs and fibroblast sub-clones contained comparable numbers of de novo variants.

### 4.2. Reprogramming-Induced Genetic Variations

Cellular reprogramming is a rare, multi-step process, which shares many biological and molecular pathways with tumorigenesis [55]. Tumor suppressor genes, including those involved in DDR, may have an inhibitory effect on nuclear reprogramming, suggesting that the process of reprogramming could lead to an elevated mutational load in hiPSCs. Accordingly, for hiPSCs deletions of tumor suppressor genes were primarily associated with reprogramming [37]. Several studies on miPSC generation highlighted that DNA DSBs arising during the process of reprogramming are the primary source of genetic instability [18,19,24,56]. However, variations with allele frequencies of about 50% cannot be distinguished whether they are pre-existing variants or reprogramming-induced mutations occurring before the first cell division. Any parental cell with a monoallelic variation will produce iPSCs with 50% allele frequencies of this pre-existing variation; at the same time, mutations induced by reprogramming may occur before the first cell division soon after the onset of cell reprogramming and will again lead to about 50% mutated allele frequencies.

Even if genetic variations are observed during reprogramming, the process itself tends to fail in case the cells of origin do not present a normal genotype, or aberration occurs during the transition to pluripotency. As reported in the second paragraph of the present review, data concerning hiPSC generation demonstrated that defects in the major components of HR or NHEJ machineries for DNA repair, including *ATM*, *FA*, *LIG4*, *XLF*, *DNA-PK*, *BRCA1*, *BRCA2* and *RAD51* prevent the generation of iPSCs [18,19,20,21,22,24,26,27]. This observation can be linked with the notion that reprogramming of cancerous cells is a rare event, and many attempts to have hiPSC lines able to model specific cancers failed [57]. Even if examples of successful human cancer reprogramming exists [58,59,60,61,62], in most of the cases, it has been reported to be a much less efficient process compared to not-transformed cells, and limited to a specific subset of “reprogramming permissive” genomic aberrations [57]. This aspect has recently been elucidated by Lee et al. [63] by attempting to reprogram more than 13 different acute myeloid leukemia (AML) primary samples with different genetic aberrations, obtaining no hiPSC colonies or hiPSCs not carrying the original patient sample aberration and then suggesting a selection for normal genomes during the reprogramming process. The only case of successful reprogramming carried a mixed lineage leukemia (MLL) aberration, suggesting the MLL family as one of reprogramming permissive aberrations as the only other cases of AML reprogramming [61,62] reported aberrations or mutations affecting this protein family, too.

Collectively, these data highlight that an efficient DDR pathway activation has a central role in preventing the reprogramming of cells with abnormalities, avoiding instable or aberrant genomes to reacquire pluripotency.

### 4.3. Culture-Induced Genetic Variations

During iPSC in vitro culturing, adaptation to culture conditions and clonal selection during passaging are common events; probably, these are the main causes of accumulation of genomic instability in iPSCs. The data reported by Taapken et al. [41] suggested that the types and frequency of karyotypic abnormalities are similar between hESCs and hiPSCs, supporting this idea. However, differences between hESC and hiPSCs sub-chromosomal variations have been revealed by Laurent et al. [37]. Interestingly, they showed that, while the reprogramming process was associated with deletions of tumor-suppressor genes, prolonged time in culture was associated with duplications of oncogenic genes.

Mayshar et al. also reported that a gain of the p region of chromosome 12, previously acknowledged in this review as the most recurrent chromosome involved in iPSC chromosomal instability, was caused by prolonged culture [34]. It is known by now that genetic stability cannot be guaranteed upon extensive in vitro passaging. During the adaptation to culturing, acquisition of growth advantage-promoting copy number aberrations cause a few hESCs to overwhelm the cell population with an obvious parallel with malignant transformation [64]. Chromosomes 12, 17 and X were found to be most commonly involved in such mutation events [64,65].

As already cited, recurrent CNVs at 1q31.3 and 17q21.1 were identified as unique to the hiPSC samples (>25% of hiPSC samples) [40]. Because they were not detected in the parental fibroblasts they seemed to have originated from genetic instability during the culture of hiPSC lines [40]. Ji et al. estimated the proportion of coding mutations in iPSCs at passages 6 and 12, that were likely acquired during passaging since the picking of the initial iPSC colony [44]. They found that coding point mutations largely persisted during passaging, as 56 of the 59 mutations in the p6-iPSC were present in the p12-iPSC [44]. Several data support the hypothesis that hiPSCs lose their repair capacity over multiple passages in vitro: Laurent et al. [37] analyzed the basal frequency of CNVs over passages and reported that pluripotent stem cells had more CNVs in comparison to non-pluripotent cells and some of these increased during passaging. Considering the repair capacity of irradiated hiPSCs, it has been reported that iPSCs maintained for long time in culture decreased their DSB repair capacity [29]. Similar results were published by Zhang et al. on one mouse iPSC line [66]. They confirmed the compromised DNA damage repair capacity of iPSCs compared with the respective source cells after γ-irradiation treatment but did not focus on the length of the in vitro culturing of iPSCs. Hussein et al. arrived to a slightly different conclusion, observing that most de novo-formed CNVs are present in early-passage hiPSCs, while fewer CNVs are found in late-passage hiPSCs and fibroblasts [36].

Taken together, these data suggest that in vitro culturing decreases DNA damage repair efficiency and contributes to hiPSC genomic instability, analogously to what observed in hESCs. Though low-passage hiPSCs show a highest degree of genomic instability if compared with high-passage cells, it cannot be excluded that some mutations conferring growth-advantage, such as those occurring in cell cycle genes, can be maintained and become fixed in the population [5].

## 5. Phenotypic Consequences of Genetic Variations in iPSCs

A big issue is whether some of the described genetic variations could increase disease risk when hiPS-derived cells/tissues are used in the clinic.

The functional consequences of genetic variations need to be carefully interpreted because it is difficult to distinguish driver mutations that can contribute to cancer development conferring a proliferative advantage from passenger mutations having virtually no effect on the fitness of a cancer clone. Though exploration of cancer genomic data might provide an insight into the effect of genetic variations observed in iPSCs, however, even when cancer mutations are found in iPSCs it does not directly mean that these will lead to tumorigenesis.

Laurent et al. highlighted increased sub-chromosomal CNVs in hiPSCs, with localization in specific genomic regions, together with deletion in tumor-suppressor genes and duplication of oncogenes in long term cultured hiPSCs [37]. Gore et al. pointed out that most protein-coding mutations in iPSCs are non-synonymous, nonsense, or splice variants, and accumulate particularly in oncogenic pathways as they are enriched in cancer-associated lists like Catalogue of Somatic Mutations in Cancer [33]. Mayshar et al. [34] performed a functional analysis of the expressed genes in recurring aberrant chromosomal regions in hiPSCs employing the DAVID Functional Annotation tool [67,68]. Annotations referring to cell cycle related genes resulted to be significantly enriched in the chromosomal regions gained in multiple (at least two) hiPSC lines, while control chromosomal regions with comparable size did not show such an enrichment [34].

On the other hand, when Ruiz et al. generated iPSCs that carry several protein-coding mutations identified in iPSCs to assess their functional effects, they found that these mutations did not generally facilitate the acquisition of pluripotency and were not likely to provide a selective advantage for reprogramming [45]. Another study demonstrated that SNVs, genetic alterations largely found by high-throughput NGS analyses in both coding and non-coding regions in iPSCs [33,35,44,45,52], are not enriched in cancer-associated gene lists [35]. It is still unclear if the protein-coding mutations in iPSCs will ever result in an altered cellular or oncogenic phenotype [69]. More recently, comparative genomic analysis was performed on nine iPSC lines generated from the same parental fibroblast cell lines with three different reprogramming methods (integrating retroviral vectors, non-integrating Sendai virus and synthetic mRNAs) [70]. Through these analyses, authors identified SNVs, insertions and deletions, and structural variants; however, all these variants were generally benign [70].

Interestingly, thanks to sufficiently large collections of iPSCs with corresponding genotype and gene expression data, genotype-expression association studies are now available. De Boever et al. used whole-genome sequencing and gene expression profiling of 215 hiPSC lines from different donors to identify genetic variants associated with RNA expression for almost 6000 genes. They identified variants that were associated with gene expression and altered transcriptional factor binding. They also observed that many common CNVs associated with gene expression levels are in intergenic regulatory regions and rare genic CNVs have relatively large effects on gene expression that can be positive or negative depending on their location relative to the gene. Finally, rare promoter SNVs overall have a small negative effect on gene expression. This study highlights that understanding the effect of genetic background on iPSC gene expression is critical for estimating pluripotency and differentiation efficiency, studying gene dysregulation in disease, and comparing differentiated tissues to somatic tissues [71].

A call for placing more consideration on examining genetic instability in iPSCs potentially leading to functional consequences is unavoidable today, in light of the robust knowledge of in vivo oncogenic phenotypes produced by defined series of mutations.

## 6. Towards Production of Genetically Stable iPSCs

Methods for maintaining the genetic stability of iPSCs are crucial for potential clinical applications [39] and many attempts have been made so far to produce iPSCs not only more efficiently, but also more safely.

### 6.1. Culture Time Reduction

The reprogramming process lasts a certain amount of time and the subsequent cell expansion and characterization steps further prolong this timeframe. Thus, the length of in vitro culturing time cannot be reduced as much as it might be necessary to limit genetic instability. Although some deleterious CNVs and DSBs that occur at earlier passages could be negatively selected and lost during subsequent passages [29,36], several studies reported that aneuploidies [34,72], CNVs [37,72], and point mutations [33] accumulate at later passages. Another aspect that must be taken into account in determining the optimal culture time concerns the effect of prolonged culture on differentiation potential. A study performed on miPSCs highlighted that miPSCs generated from different somatic cell lines differ in their differentiation potential [73]; these differences correlate with different histone and DNA methylation profiles related to the tissues of origin. The specific methylation profiles limiting differentiation potential disappear during successive cultures [73]. In this way, the time of culture should not be too short to pose the risk of biased differentiation potential nor too long to induce the accumulation of DNA aberrations This suggests that further studies are required to determine the optimal passage number for clinical applications and that careful monitoring will remain crucial to ensure iPSC safety in clinical use, though currently there are no evidence-based guidelines for tumorigenicity testing of iPSCs. Genomic and functional evaluation of iPSCs (besides epigenomic stability assessment, another fundamental issue not addressed in this review) would be essential with the advent of newer iPSC generation protocols.

### 6.2. Reprogramming Methods

An important issue related to reprogramming-induced genetic variations is the reprogramming method used. As previously discussed, all the reprograming methods that involve genome-integrating DNA elements cause genetic abnormalities in the generated iPSCs [36,37,38,40]. For this reason, researchers made a great effort to identify efficient and safe reprogramming methods avoiding genomic perturbation. The use of episomal vectors [74,75], adenoviral vectors [76], Sendai viral vectors [77], expression plasmids [78], synthetic mRNA [79], miRNA [80], protein transduction [81,82] and small molecules [83] led to good results, in terms of possibility to generate functional iPSCs. However, all these procedures present some limitations, including low reprogramming efficiency, serial transgene delivery requirement, or viability to only certain types of somatic cells. However, considering that genomic stability is critical for clinical applications of hiPSCs, it is relevant to evaluate if these methods could confer an increased genomic stability. Kang et al. investigated reprogramming method-specific genomic aberrations in a high-resolution microarray platform that enables the detection of kilobase-length aberrations [84]. They demonstrated that non-integrating iPSC lines display lower level of CNV. According to these results, using episomal vectors may lower the risk of reprogramming-associated genome changes [35,84]. More than 1000 heterozygous SNVs were found in hiPSC lines induced even by non-integrating plasmid expression, half of which being situated within coding regions in each iPSC line [35], however, ~50% of these SNVs were synonymous changes and the remaining were not selectively enriched for known genes associated with cancers.

These data thus suggested that not-integrating cell reprogramming minimize the genomic instabilities of iPSCs, [85]. However, iPSCs generated with protein-based method were still found to carry many de novo genetic variants [86], indicating that other parameters in addition to reprograming methods have to be considered to optimize genomic stability in iPSCs.

### 6.3. Reprogramming Factors

A better understanding of the reprogramming factors is nowadays largely explored to generate safer iPSCs. As oncogene activation is a major driver of genomic instability, Pasi et al. questioned the genomic status of iPSCs generated by overexpression of the four classical factors *Oct4*, *Sox2*, *Klf4* and *c-Myc*, all having well established roles in tumorigenesis [55]. The negative effect of c-Myc on the occurrence of genomic abnormalities, including deletions and amplifications, during reprogramming has been demonstrated by Pasi et al. through analysis of CNVs in iPSCs generated either in the presence or in absence of c-Myc [87]. Considering that c-Myc is dispensable for iPSC generation [88,89] and its relevant negative effects on genomic stability [87], it is safe recommending to omit c-Myc in generating hiPSC for clinical applications. Notably, currently, there are known replacements for each of the four classic reprogramming factors (reviewed in [90]).

Other factors in addition to the classic reprogramming ones may also increase genomic stability during reprogramming. It was demonstrated that p53 plays a dual role in iPSC reprogramming [91]. Early reprogramming of iPSCs induced by Yamanaka factors triggers the DDR that activates p53. It is well known that p53 activation prevents the reprogramming of cells carrying unresolved DNA damage by activating apoptosis or senescence of these cells [28,92,93]. Consequently, in the absence of p53, reprogramming efficiency increases significantly, but the generated iPSCs have a high risk of carrying genetic aberrations [28,94]. One solution to improve iPSCs genetic quality is the overexpression of truncated p53 truncated form Δ133p53 during reprogramming. This isoform has a truncated N-terminal (transcribed by an alternative p53 promoter in intron 4) and is a p53 target gene able to antagonize p53 mediated apoptosis [95] and to promote DNA DSB repair [96]. As confirmation, recently it has been shown that Δ133p53 is induced upon reprogramming and overexpression of Δ133p53 with the four classical factors Oct4, Sox2, Klf4 and c-Myc significantly increases reprogramming efficiency together with a decrease in chromosomal aberrations. [97].

Further, employment of oocyte factor Zscan4 in combination with the Yamanaka factors during iPSC reprogramming was found to stabilize the somatic genome resulting in improved quality of the induced cells, in addition to improving reprogramming efficiency itself [56] More recently, it was found that increasing the levels of the checkpoint kinase 1 (CHK1) reduced the replication stress associated with reprogramming and simultaneously improved the efficiency of iPSC generation [98].

### 6.4. Oxidative Stress Reduction

Culture conditions play an important role in regulating the genomic integrity of iPSCs. Currently, different substrates and media are available to support feeder-free cultures of hiPSCs. A recent study investigated if the metabolic activity of hiPSCs in different media could impact genetic integrity of hiPSCs [99]. Authors showed that, compared to cells cultured in Knockout Serum Replacement media (KSR), hiPSCs kept in two widely used media for feeder-free culturing, E8 and TeSR, showed dramatically increased ROS levels and higher mitochondrial potential, associated with increased levels of markers for DSBs and increased radiosensitivity. Resulting cells were demonstrated to possess an altered genomic status: the number of SNVs in hiPSCs cultured in E8 and mTeSR increased 1.7 times and 3 times, respectively, when compared to KSR.

Noteworthy, oxidative stress could be reduced by hypoxic culture condition (3–5% O_2_), preventing DNA mutation accumulation and differentiation as well as promoting cell survival. Additionally, hypoxia was shown to accelerate the metabolic switch required for acquisition of pluripotency, enhancing the efficiency in iPSC generation [100,101]. Several evidences suggested the possibility that the genetic integrity of iPSCs may be enhanced by hypoxia, protecting cells from oxidative stress and DNA damage during reprogramming. In fact, antioxidant agent ascorbic acid improved reprograming efficiency [102], use of antioxidants to lower ROS levels improved iPSCs quality[103] and the addition of either one or two antioxidants (*N*-acetyl-cysteine and ascorbic acid) during reprogramming, was able to reduce ROS levels and generate iPSCs with less de novo CNVs [103]. MTH1, OGG1, and MUTYH are the main enzymes involved in base excision repair pathway, preventing mutations associated with 8-oxoguanine, a common product of oxidative damage to DNA [104]. In this way, it will be interesting to examine whether overexpression of these enzymes in the cells during reprogramming will help to preserve the genome integrity of iPSCs.

## 7. Conclusions

The data collected in this review highlight the importance of understanding the molecular mechanisms underlying genomic instability during reprogramming process.

Cell reprogramming, for its intrinsic characteristics, involves DNA damage response activation; however, another intrinsic characteristic of cell reprogramming is to avoid reprogramming occurrence in the presence of dangerous genetic anomalies. Data accumulated so far on the genetic stability of pluripotent stem cells suggest that in vitro culturing affects in a similar way both ESCs and iPSCs, but also highlight that efficient repair mechanisms are needed to allow successful cell reprogramming. The reason for different DDR pathway involvement in cell reprogramming is likely to avoid presence of aberration from the process itself or from the cells of origin.

Meanwhile, it is equally important to characterize and classify the genomic aberrations acquired by iPSCs to discriminate adverse from inconsequential abnormalities.

Much effort today is dedicated to optimization of reprogramming and culture conditions to improve genetic stability of iPSCs and their safety for clinical cell therapy. Further rigorous work on mutation rates and distributions during in vitro culture and reprogramming of iPSCs will be essential to help establish clinical safety standards for genomic integrity.

## Figures and Tables

**Table 1:** Genetic variants identified in hiPSCs.

Genetic Variants	Described Changes	References
Chromosomal Instability	Trisomy 12	[34,37,40,41]
	Trisomy 20q	[41]
	Trisomy X	
	Trisomy 8	
Copy Number Variations	Amplification of 20q11.21	[37,40,42,43]
	Amplification of 1q31.3	[32,40]
	Deletion of 17q21.1	
	Deletion of 8q24.3	
	Duplication of 2p11.2	[42]
Single Nucleotide Variations	22 hiPSC lines derived from 7 fibroblast cell lines:	[33]
	124 single nucleotide mutations (missense, nonsense, splice variants) identified.	
	6 mutations per iPSC genome on average.	
	8 mutations described in more than 1 cell line (*OR52E8*, *SLC1A3*, *MYRIP*, *HK1*, *ANKRD12*, *SCN1A*, *NEK* family genes, *NTRK* family genes).	
	5 hiPSC lines derived from 1 fibroblast cell line:	[44]
	59 single nucleotide mutations (missense, nonsense, splice variants) identified.	
	12 mutations per iPSC genome on average.	
	10 mutations described in more than 1 cell line (involved genes not specified).	
	8 iPSC lines derived from 4 different somatic cell types (neural stem cells, astrocytes, umbilical vein endothelial cells, foreskin keratinocytes):	[45]
	40 single nucleotide mutations (missense, nonsense, splice variants).	
	5 mutations per iPSC genome on average.	

## Abbreviations

iPSCs	Induced Pluripotent Stem Cells
ESCs	Embryonic Stem Cells
DDR	DNA Damage Response
DSB	Double Strand Break
ROS	Reactive Oxygen Species
HR	Homologous Recombination
LIG4	DNA Ligase IV
NER	Nucleotide Excision Repair
NHEJ	Non-Homologous-End-Joining
CNVs	Copy Number Variations
SNVs	Single Nucleotide Variations
CGH	Comparative Genomic Hybridization
AML	Acute Myeloid Leukemia
MML	Mixed Lineage Leukemia
XP	Xeroderma Pigmentosum
